# Analysis of the Strength Properties of Epoxy–Glass Composites Modified with the Addition of Rubber Recyclate Using Kolmogorov–Sinai Metric Entropy

**DOI:** 10.3390/ma17020411

**Published:** 2024-01-13

**Authors:** Daria Żuk, Norbert Abramczyk, Grzegorz Hajdukiewicz

**Affiliations:** Faculty of Mechanical Engineering, Gdynia Maritime University, 81-225 Gdynia, Poland; n.abramczyk@wm.umg.edu.pl (N.A.); g.hajdukiewicz@wm.umg.edu.pl (G.H.)

**Keywords:** epoxy–glass composites, rubber recyclate, static tensile test, Kolgomorov–Sinai metric entropy

## Abstract

This paper presents the results of investigations of the mechanical properties of epoxy–glass composites with the addition of rubber recyclate. For the purposes of the study, seven variants of materials were designed and manufactured, which differed in terms of the percentage of recyclate content (3, 5 and 7%) and the way the recyclate was distributed in the composite (one, two and three layers with a constant share of 5%). Tests of comparative mechanical properties were carried out using a static tensile test. As a result of the conducted tests, the following values were obtained for all variants of materials: tensile strength (*R_m_*), Young’s modulus (*E*) and percentage relative strain *ε*. In addition, for a deeper analysis of the results obtained, statistical calculations of Kolgomorov–Sinai *E_K-S_* metric entropy were performed on the experimental data sets, which were then analyzed. The results of the analysis indicate that the application of metric entropy calculations *E_K-S_* can be helpful in identifying changes in the internal structure of the composite material that occur during its loading, and which do not manifest themselves in any other tangible way. The data obtained as a result of the research can be used to optimize production processes and to determine the further direction of development of epoxy–glass composites with the addition of rubber recyclate, while saving time and resources.

## 1. Introduction

In recent decades, advances in materials science have significantly contributed to the development of modern composites, which are used in many key industrial sectors [1,2]. One such material is epoxy–glass composites, which are valued for their exceptional mechanical and thermal properties.

In recent years, there has been a significant increase in interest in composites based on glass mats and epoxy resins [1,2,3]. This is due to their unique mechanical properties, which predestine these materials for use in many industries, such as construction, automotive, or aerospace [1,2]. Although these composites are already widely used, intensive research is underway, aiming to improve their strength and resistance to various types of loads [3,4].

In addition, the growing environmental awareness and the need to recycle industrial materials prompts the search for innovative methods of their use. In this context, the addition of rubber recyclate to epoxy–glass composites represents a promising direction of research aimed not only at improving mechanical properties, but also at influencing sustainability and ecology.

The contemporary development of composite technologies opens up new possibilities for the use of recycling materials, such as rubber recyclate, to improve the performance and environmental properties of traditional composites [5,6,7,8,9]. Composites based on glass mats with the addition of rubber recyclate are an interesting area of research due to the potential to combine the properties of these two materials. In the literature, one can find analyses of the influence of modifiers on the properties of epoxy–glass composites in terms of their strength parameters such as tensile strength, impact strength, or modulus of elasticity [7,10,11]. These analyses also apply to the addition of rubber recyclate and rubber materials in composites [12,13,14,15,16,17,18].

Metric entropy [19,20,21] is a concept derived from information theory and mathematical chaos theory used to describe the complexity of dynamical systems. It is a measure of the disorder of the system. In the context of physics and materials science, metric entropy can be used to preserve materials under load, especially materials with complex, irregular structures (anisotropic materials).

In the study of epoxy–glass composites modified with rubber recyclate, metric entropy allows for a deeper understanding and interpretation of the impact of microstructural changes on the mechanical and strength properties of the material subjected to forces.

In order to obtain a more complete picture of the material’s behavior under load, the statistical method of metric entropy calculations, which illustrates the change in the dynamics of material deformation during the test, was used. The use of Kolgomorov–Sinai metric entropy calculations to analyze the strength properties of materials is a relatively new tool [19,20]. Studies [19,20,21,22,23,24] have shown that with the use of modern testing machines, the Kolmogorov–Synai metric entropy method (K-S) and the acoustic emission (AE) method, it is possible to determine the transition from the elastic phase to the plastic phase for a composite material. Properly processed experimental data sets (in this article, the *E_K-S_* calculations were performed on sets of *ε* values) allow us to capture the changes occurring in the structure of the material (including the internal structure) under the influence of loading. This method assumes that the qualitative changes occurring in the structure of the material correspond to a specific measuring point. This point is critical and separates the phases of elastic deformations and elastic–plastic deformations. The application of this method in the testing of the strength properties of epoxy–glass–rubber composites is an attempt to determine the value of stresses up to which this material can be safely exploited.

This article focuses on the analysis of the strength properties of such modified composites, using a novel method based on Kolgomorov–Sinai metric entropy. This unique method allows for an in-depth understanding of the impact of the addition of rubber recyclate on the structure and properties of composites, offering new perspectives in the design of high-strength materials. By combining the theoretical foundations of metric entropy with the practical aspects of materials engineering, the article aims not only to contribute to the development of materials science, but also to indicate potential paths for industrial applications of such composites. The next part of the article will focus on the research methodology, experimental results and their analysis, striving to fully understand and exploit the potential of the modification of epoxy–glass composites with the addition of rubber recyclate.

## 2. Materials and Methods

### 2.1. Properties of the Rubber Recyclate Used

Rubber recyclate is produced by recycling used car tires. Tires contain a mix of different materials, such as rubber, steel and fabrics. Rubber recycling aims to recover and reuse these materials to reduce waste and reduce the need for new raw materials.

Orzel-Base rubber recyclate from industrially processed car tires with a grain size of 0–3 mm was used as a modifier for the study. The recyclate was sieved using a laboratory sieve shaker LAB 11-200 from EKOLAB in order to precisely separate its fraction. As an additive to the epoxy resin, a fraction of recyclate with a grain size of 0.5 mm to 1.5 mm was used in the composite. The percentage composition and physico-chemical parameters of the rubber recyclate used in the tested materials are presented in Table 1 and Table 2. 

### 2.2. Production of Research Materials

All variants of the designed materials were manufactured with the use of the EM1002/300/125 construction glass mat, with a random distribution of fibers and a weight of 350 g/m^2^. The composite was based on epoxy resin Epidian@ 6 with hardener Z-1 (Table 3). The base material was the *K0* composite (without the addition of recyclate). The research materials were *L3*, *L5* and *L7* composites (with different amounts of recyclate added, which were directly mixed with resin), as well as *K1*, *K2* and *K3* composites (with 5% recyclate content in the composite and variable decomposition in its layers). A detailed list of the content of components in all variants of the produced composites is presented in Table 4.

**Figure 1:**
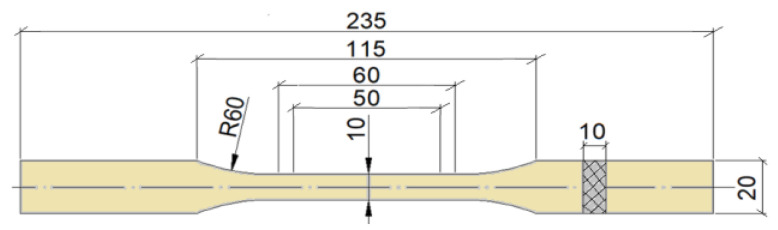
Geometry of test specimens.

**Figure 2:**
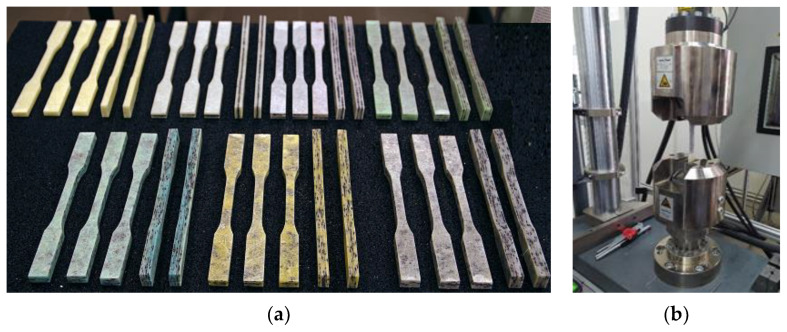
(**a**) List of all variants of test materials; from top left: *K0*, *K1*, *K2*, *K3*, *L3*, *L5*, *L7*; (**b**) *K2* composite sample during static tensile testing.

The research materials were made using manual lamination with the use of constant double-sided pressure for all variants. The value of the pressure (675 N per mold) has been determined in such a way as to prevent excessive filtration and leakage of the resin. A 300 × 900 mm cuboidal steel mold, brushes and rollers were used for manual lamination. A static tensile test was performed for samples prepared via waterjet cutting in seven material variants and 10 pieces for each variant in accordance with the PN-EN ISO 527-4: 2022-06 standard [26]. Figure 1 shows the geometry of the samples. The cut samples were subjected to a static tensile test on a hydraulic testing machine of the Zwick and Roell type MPMD P10B with testXpert II v 3.61. Figure 2a shows samples made from all variants of the materials produced and tested. Figure 2b shows a sample of *K3* composite during site testing.

## 3. Results and Discussion

### 3.1. Static Tensile Test

The obtained measurement results were used to perform a further comparative analysis of the tested material variants in terms of the strength parameters presented in Table 5 and allowed us to determine the effect of rubber recyclate on these parameters. The obtained measurement results allowed us to perform a detailed analysis and to distinguish variants of materials with the most favorable strength parameters.

**Figure 3:**
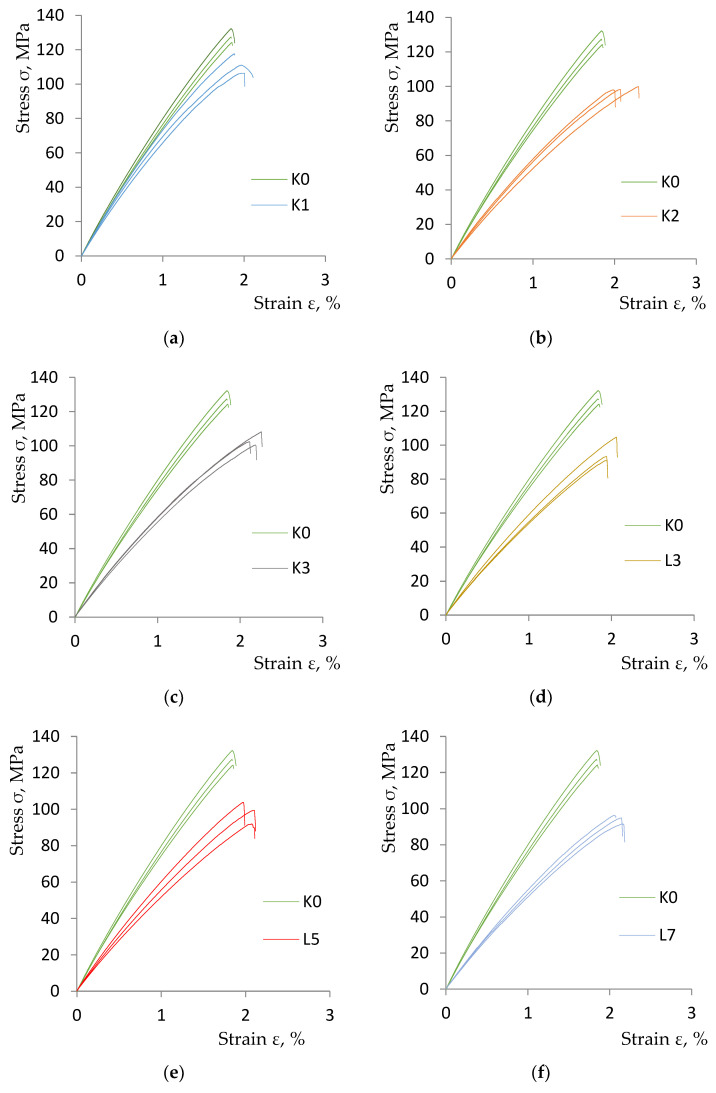
Diagrams of the static tensile test of the tested variants of composite materials obtained from the Zwick Roell testing machine: (**a**) *K0* and *K1* specimens; (**b**) *K0* and *K2* samples; (**c**) *K0* and *K3* samples; (**d**) *K0* and *L3* samples; (**e**) *K0* and *L5* samples; (**f**) Samples *K0* and *L7*.

Figure 3 presents a summary of the diagrams obtained during the static tensile test for three randomly selected measurements of all analyzed material variants (*K1*, *K2*, *K3*, *L3*, *L5*, *L7*) in relation to the comparative material *K0*. A summary of the parameters obtained from TestXpert II version 3.61 composite materials is presented in Table 5.

The addition of rubber recyclate to epoxy resin-based composites and glass mats can have a significant impact on their strength properties. As the research has shown, the addition of rubber recyclate reduces the stiffness of the composite, which leads to a decrease in the tensile strength for all tested material variants. An important issue is the interaction between the rubber recyclate and the resin–glass matrix. If the adhesion between these components is poor, it can lead to the formation of microcracks and structural weakening of the composite. Therefore, it is essential to optimize the mixing and curing process to ensure good adhesion between the recyclate and the resin. The addition of rubber recyclate to epoxy resin-based composites and glass mats can lead to significant changes in their strength properties. The final result depends on a number of factors, including the proportion, size and type of recyclate, as well as the method of preparation and processing of the composites. Therefore, it is crucial to conduct detailed research and testing to optimize the composition and manufacturing process for specific applications.

### 3.2. Metric Entropy Analysis of the Mechanical Properties of Individual Material Samples

In order to evaluate the structural complexity analysis of the composites, Kolgomorov–Sinai metric entropy was used to compare the entropy profiles for the different applied proportions of recyclate in the composites and to understand how the modification affects the disorder and irregularity of the composite structure.

Using entropy analysis to interpret stress test results offers a deeper insight into the relationship between structure and mechanical properties. This methodology allows for a comprehensive study of the effect of the addition of rubber recyclate on the strength properties of epoxy–glass composites and allows us to understand this impact from the perspective of both traditional strength-testing methods and advanced metric entropy analysis.

**Figure 4:**
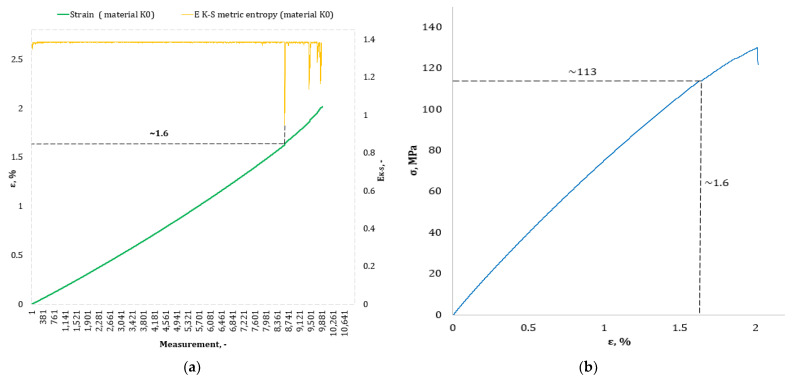
(**a**) The course of the strain curve for the *K0* composite sample; (**b**) the results of the metric entropy calculations *E_K-S_* as a function of the test time for the *K0* composite sample.

**Figure 5:**
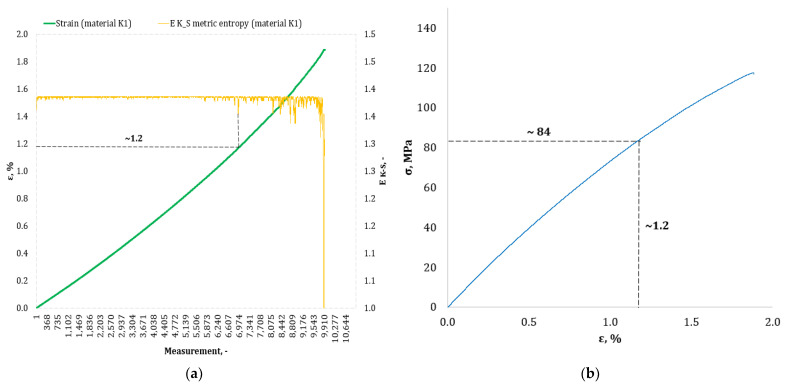
(**a**) The course of the strain curve for the *K1* composite specimen; (**b**) the results of the calculation of metric entropy *E_K-S_* as a function of test time for the *K1* composite sample.

**Figure 6:**
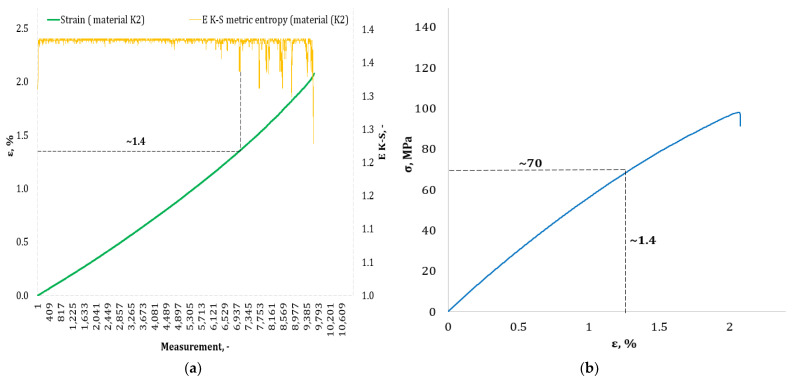
(**a**) The course of the strain curve for the *K2* composite specimen; (**b**) the results of the calculation of the metric entropy *E_K-S_* as a function of the test time for the *K2* composite sample.

**Figure 7:**
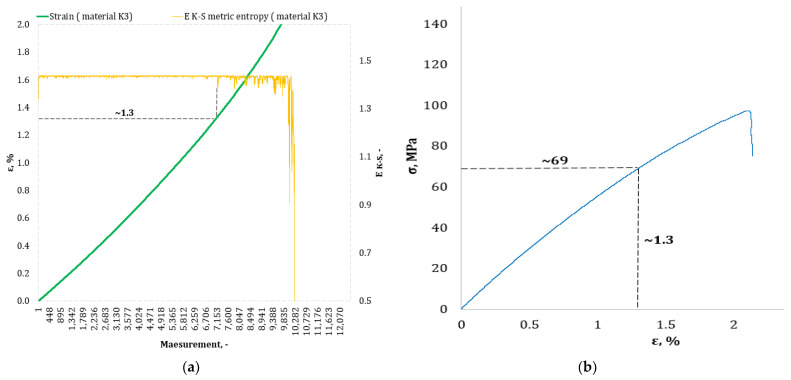
(**a**) The course of the strain curve for the *K3* composite specimen; (**b**) the results of the calculation of the metric entropy *E_K-S_* as a function of the test time for the *K3* composite sample.

**Figure 8:**
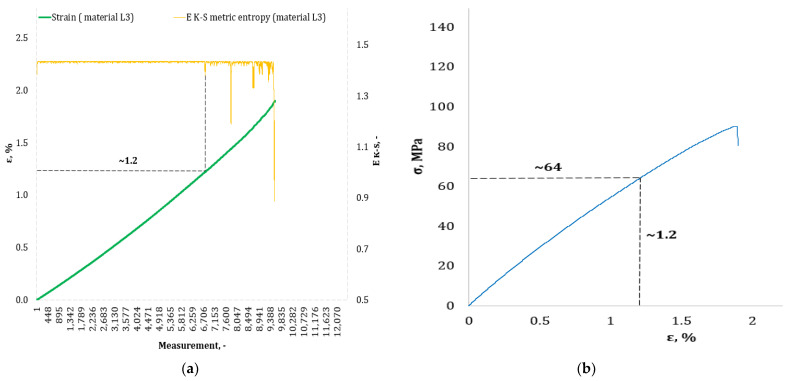
(**a**) The course of the strain curve for the *L3* composite specimen; (**b**) the results of the calculation of metric entropy *E_K-S_* as a function of test time for the *L3* composite sample.

**Figure 9:**
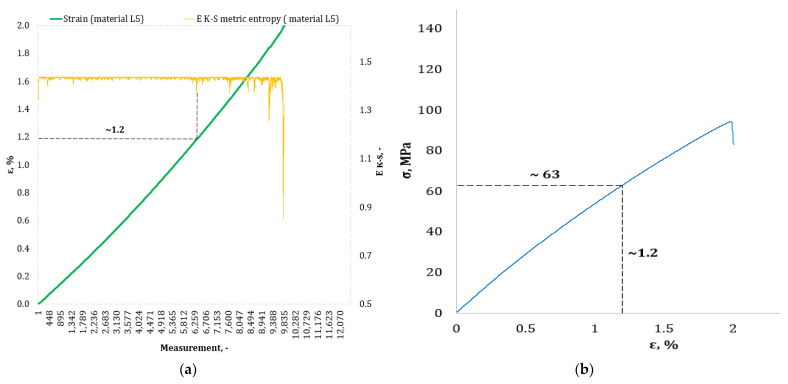
(**a**) The course of the strain curve for the *L5* composite specimen; (**b**) the results of the calculation of metric entropy *E_K-S_* as a function of the test time for the *L5* composite sample.

**Figure 10:**
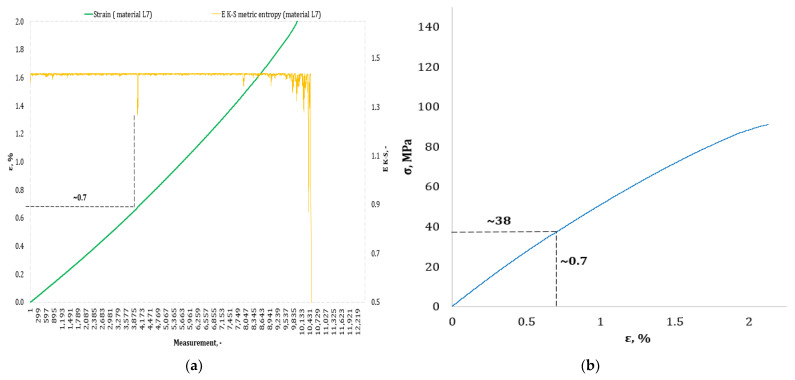
(**a**) The course of the strain curve for the *L7* composite specimen; (**b**) the results of the calculation of the metric entropy *E_K-S_* as a function of the test time for the *L7* composite sample.

Figure 4, Figure 5, Figure 6, Figure 7, Figure 8, Figure 9 and Figure 10 show selected tensile curves that represent each type of material used in the test. These drawings consist of part (a), which shows the course of the *ε* strain curve, and part (b) showing the results of calculations of metric entropy *E_K-S_* as a function of the study time *ε* = *f*(*t*). The time represented is the number of the next (discrete) measurement point. The test was performed with the testing machine sampling set to 50 Hz, so 1 s of the test corresponds to 50 consecutive measurement points. Calculations of metric entropy *E_K-S_* were performed on a set of *ε* values for each tested sample maintaining the same lengths of the assumed intervals, i.e., 40 measurement points, and the same number of adopted sub-intervals, i.e., four. The calculations were performed using the proprietary Entropy K-S program version 1.20. The methodology of metric entropy calculations used in this study is described in detail in the source items [19,20,22] where phase portraits of metric entropy are additionally presented.

For each variant of the composite material under study, a characteristic point for which the entropy value decreases was indicated in the course of changes in metric entropy *E_K-S_*. Such a reduction in the calculated metric entropy value illustrates a change in the dynamics of the phenomenon. At this point in the test, the composite specimen corresponds to a dynamic change of deformation into a uniformly increased tensile force. This point corresponded to a critical qualitative change taking place inside the composite. This change may correspond to one or more phenomena that initiate irreversible degradation of the material. These phenomena are the onset of matrix cracks, delamination of composite fibers, and delamination of additives (including rubber recyclate). After indicating the number of the measuring point which corresponded to the decrease in the value of the calculated metric entropy *E_K-S_*, we read the strain value *ε_K-S_* for this measurement point. Then we transferred the read value of the strain *ε_K-S_* from parts (a) of Figure 4, Figure 5, Figure 6, Figure 7, Figure 8, Figure 9 and Figure 10 to part (b) of these drawings, i.e., the diagram *σ* = *f*(*ε*) obtained from the testing machine, and read the corresponding value of stresses σ*_K-S_*. 

Table 6 compares the mean values of *σ_m_* and *ε* obtained in the static tensile test and the mean values of the relative strain *ε_K-S_* and the corresponding stress values *σ_K-S_* obtained via the Kolgomorov–Sinai metric entropy calculations *E_K-S_*.

Analyzing the results presented in Table 6, we can see that in the case of each type of epoxy–glass composite produced, we are dealing with critical, qualitative changes occurring in its structure before reaching the tensile strength value, i.e., *σ_m_*. This is the case both in terms of a pure (*K0*) composite without the addition of recyclate, which obtained an average value of *σ_m_* = 127.3 MPa in a static tensile test, and an average value of *σ_K-S_* = 113 MPa which was recorded using metric entropy calculations, i.e., a value lower by 11%. Other types of epoxy–glass materials into which rubber recyclate was introduced showed a greater reduction in the value of stresses at which significant internal damage occurs in their structure. For the *K1* material, where the recyclate was placed in the form of one layer, the reduction of the value of *σ_K-S_* = 84 MPa in relation to the tensile strength *σ_m_* = 108.4 MPa was 23%. Other types of epoxy–glass material *K2*, *K3*, *L3*, *L5* with rubber recyclate showed a more than 30% decrease in the value of stresses at which critical qualitative changes occur in the structure in relation to the tensile strength *σ_m_* of these materials. As far as the values of deformations at which a decrease in the metric entropy value was recorded, for the base material, i.e., epoxy–glass composite without *K0* recyclate, the average value *ε_K-S_* was 1.6% and was 14% lower than the relative elongation at break *ε* = 1.86% for this material. Mean relative elongation *ε_K-S_* reduced the metric entropy value of samples containing rubber recyclate from 1.2% to 1.4%. The exception was *L7* samples with a randomly arranged recyclate in the structure in the amount of 7%. In the case of this material, the decrease in the mean values *ε_K-S_* = 0.7% compared to the value of *ε* = 2.11% reached 67%. In the authors’ opinion, such a large decrease in the value of *σ_K-S_* in relation to *σ_m_* and *ε_K-S_* in relation to *ε* for this type of composite was due to the percentage of rubber recyclate. This material was the only one that contained as much as 7% of the rubber component in the form of granules. The rubber recyclate was placed randomly (not in layers) in the structure of the material, which, with such a large amount of crushed rubber, critically affected its mechanical properties. Due to the random distribution of the recyclate, the results of the strength parameters may be irregular. In addition, increasing the percentage of recyclate will increase the range of irregularities in the results.

Summarizing the analysis of strength properties performed by means of metric entropy calculations for all seven types of composites studied, it was noted that the values of critical stresses *σ _K-S_* at which changes occur in the structure of composites are lower than the value of tensile strength of these composites. This means that for each type of test material (including the starting material without the addition of rubber recyclate), load limits corresponding to these critical stresses should be adopted in its possible operation. Using the *E_K-S_* calculations, it was also noted that the addition of rubber recyclate significantly changes the elastic plastic properties of the composite. In each case, a decrease in the value of the *ε_K-S_* was recorded. Taking into account the percentage values of reduction of *σ_K-S_* in relation to *σ_m_* and *ε_K-S_* in relation to *ε*, it should be considered that the composite with the addition of rubber recyclate in the form of one layer, i.e., the *K1* variant, loses the least in terms of strength in relation to the starting material. 

## 4. Conclusions

The results in Table 6 show that the strength properties of sandwich composites obtained in the static tensile test, such as *σ_m_* and *ε* (*ε_m_*), do not clearly describe this type of material. In particular, they do not unambiguously describe composite materials modified with additives, which has been proven using other types of tests such as acoustic emission or optical techniques for measuring surface deformations [21,27,28]. The calculations of metric entropy *E_K-S_* performed on sets of elongation values obtained in the static tensile test (a test taking place at a constant rate of force build-up) allowed us to perfectly illustrate the dynamics of the material destruction process. The use of Kolgomorov–Sinai metric entropy allowed for a deeper understanding of the structural complexity of composites. Higher entropy values for samples with a higher addition of recyclate indicated greater irregularity and disorder of the structure. 

This analysis provided new insights into the relationship between the microscopic structure and macroscopic strength properties of composite materials. Presenting the results of metric entropy calculations in graphical form as *E_K-S_ = f(t)* for each sample provides an opportunity for a broader assessment of the quality of this material. The use of *E_K-S_* calculations in the era of widespread use of very fast and relatively inexpensive calculating machines, such as modern computers, allowed us to illustrate changes in the structure of the studied materials in a way that has not been available so far. Taking into account the percentage values of reduction of *σ_K-S_* in relation to *σ_m_* and *ε_K-S_* in relation to *ε*, it should be considered that the composite with the addition of rubber recyclate in the form of one layer, i.e., the *K1* variant, loses the least in terms of strength in relation to the starting material. The most advantageous variant of the composite turned out to be the *K1* sandwich composite with one layer of recyclate in the amount of 5% of the composite’s weight. 

In further analyses of the feasibility of using the designed new material, the above-mentioned variant of the *K1* composite is recommended. 

## Figures and Tables

**Table 1:** Percentage composition of rubber recyclate [25].

Ingredient	Content, %
Natural rubber	15
SBR (Styrene–butadiene rubber)	20
BR (Butadiene Rubber)	10
IIR/XIIR (butyl rubber and halogenated butyl rubber)	5
Silica	15
Soot	15
Sulphur	2
Resin	2
Mineral and vegetable oils	10
Other (Zinc oxide, stearic acid)	6
Natural rubber	15

**Table 2:** Physical and chemical properties of rubber recyclate used in materials [25].

Parameter	Value
Density	360–370 kg/m^3^
Flash Point	>350 °C
Thermal decomposition	>180 °C

**Table 3:** Characteristics of Epidian^®^ epoxy resin 6.

Parameter	Unit	Value
Epoxy Number	[mol/100 g]	0.510–0.540
Density at 25 °C	[g/cm^3^]	1.17
Viscosity at 25 °C	[mPa∙s]	1000–1500
Gel time 100 g at 20 °C	[min]	20
Curing time at 20 °C	[days]	7

**Table 4:** Mass content of components of the tested composites.

Composite Designation	Arrangement Recyclate	Number of Layers of the Mat	Resin Content, %	The Content of the Glass Mat, %	Content Recyclate, %
*K0*	-	12	60%	40%	0%
*K1*	1 layer	12	60%	35%	5%
*K2*	2 layers	12	60%	35%	5%
*K3*	3 layers	12	60%	35%	5%
*L3*	Random in warp	12	60%	37%	3%
*L5*	Random in warp	12	60%	35%	5%
*L7*	Random in warp	12	60%	33%	7%

**Table 5:** Average strength parameters obtained from Zwick Roell’s TestXpert II 3.61 software for composite materials (measurements from 10 samples for each material variant).

Material	*σ_m_*, MPa	*ε*, %	*E*, MPa
*K0*	127.3	1.86	8446
*K1*	108.4	1.82	7946
*K2*	103.7	2.15	6418
*K3*	100.6	2.13	6255
*L3*	98.2	2.02	6198
*L5*	97.7	2.06	6066
*L7*	93.1	2.11	5829

**Table 6:** Mean values of strength parameters obtained in the static tensile test and mean results obtained using metric entropy calculations.

Material	*ε*, %	*σ_m_*, MPa	*ε_K-S_*,%	*σ_K-S_*, MPa	Changing the *ε_K-S_* Relative to *ε*, %	Change in *σ_K-S_* Relative to *σ_m_*, %
*K0*	1.86	127.3	1.6	113	−14	−11
*K1*	1.82	108.4	1.2	84	−34	−23
*K2*	2.15	103.7	1.4	70	−35	−32
*K3*	2.13	100.6	1.3	69	−39	−31
*L3*	2.02	98.2	1.2	64	−41	−35
*L5*	2.06	97.7	1.2	63	−42	−36
*L7*	2.11	93.1	0.7	38	−67	−59

## Data Availability

Data is contained within the article.
